# ASO Author Reflections: Reexamining Physical Examination in Modern Breast Cancer Survivorship

**DOI:** 10.1245/s10434-026-19399-2

**Published:** 2026-03-23

**Authors:** G. Paul Wright, Jessica L. Thompson

**Affiliations:** 1 Division of Cancer Health, Corewell Health West, Grand Rapids, MI USA; 2 Michigan State University College of Human Medicine, Grand Rapids, MI USA

## Past

Post-treatment surveillance after curative intent breast cancer treatment has traditionally emphasized regularly scheduled, in-person visits incorporating routine physical examination.^1^ These practices were established before widespread adoption of high-quality breast imaging, advances in systemic therapies, and increased patient engagement in symptom reporting. Despite growth of survivorship populations, guidelines have continued to endorse frequent follow-up visits and examinations, even as contemporary evidence defining incremental value of clinical breast and chest wall exams for recurrence detection remains limited. This knowledge gap has become increasingly relevant in the setting of workforce constraints and increasing healthcare utilization.

## Present

In this single-institution retrospective cohort study, we evaluated the diagnostic yield of provider-performed breast and chest wall examinations during survivorship follow-up. Across more than 12,000 visits involving more than 3,300 patients, physical examination accounted for a small proportion of recurrence detections. Most relapses were identified through patient-reported symptoms or surveillance imaging, while distant recurrences were most often detected through symptom-prompted diagnostic imaging rather than examination findings.^2^ These findings align with contemporary observational studies and systematic reviews demonstrating that routine physical examination contributes minimally as an independent modality for recurrence detection when compared with imaging and patient self-report.^3–5^ Collectively, these data suggest that although survivorship visits remain clinically valuable, the diagnostic contribution of routine examination alone is limited in modern breast cancer care.

## Future

These findings support reconsideration of standardized, examination-centric survivorship models in favor of more flexible, risk-adapted approaches. Future strategies should emphasize patient education regarding symptom recognition, timely access to diagnostic evaluation, and consistent use of high-quality surveillance imaging. Alternative care models incorporating telehealth, nurse-led follow-up, and patient-initiated visits may preserve clinical oversight while reducing reliance on low-yield in-person examinations. Prospective, multi-institutional studies incorporating patient-centered outcomes, recurrence risk stratification, and healthy equity considerations will be critical to informing survivorship frameworks that are evidence-based, sustainable, and responsive to the evolving needs of breast cancer survivors.
